# Citri Reticulatae Pericarpium Limits TLR-4-Triggered Inflammatory Response in Raw264.7 Macrophages by Activating RasGRP3

**DOI:** 10.3390/ijms241813777

**Published:** 2023-09-07

**Authors:** Ji Hye Lee, Yon-Suk Kim, Kang Hyun Leem

**Affiliations:** 1 School of Korean Medicine, Pusan National University, Busandaehak-ro 63beon-gil, Geumjeong-gu, Busan 46241, Republic of Korea; 2 Department of Biotechnology, College of Biomedical and Health Science, Research Institute of Inflammatory Disease (RID), Konkuk University, Chungju 27478, Republic of Korea; 3 College of Korean Medicine, Semyung University, 65 Semyung-ro, Jecheon 27126, Republic of Korea

**Keywords:** immunomodulatory effects, macrophage, RasGRP3, Citri Reticulatae Pericarpium

## Abstract

Inflammation is an important immune response to pathogen invasion, but excessive inflammation leads to tissue injury and even cytokine storm. Therefore, proper response is needed depending on the intensity of the infection. Ras guanine nucleotide releasing protein 3 (RasGRP3) is a regulator of the TLR-mediated response. In low-intensity inflammation, it negatively regulates production of pro-inflammatory cytokines, especially IL-6. Citri Reticulatae Pericarpium, the peel of *Citrus reticulata* Blanco, is a major medicinal herb in Korean medicine. The present study aims to investigate whether the Citri Reticulatae Pericarpium extract (CRE) has immunomodulatory activity using the Raw264.7 macrophage. Also, we investigated the effect of CRE on RasGRP3 expression. In the present study, CRE reduced IL-6 production in the low-LPS environment (1 ng/mL) and did not in the high-LPS environment (100 ng/mL). The suppression of IL-6 production in the low-LPS environment (1 ng/mL) was abolished after the pretreatment of RasGRP3 siRNA. The reduced RasGRP3 protein content by 100 ng/mL LPS treatment was increased by CRE treatment. Additionally, nobiletin, a major component of CRE showed a suppressive effect on IL-6 production in the low-LPS environment (1 ng/mL). The present results suggest that CRE alleviates inflammatory response via activating RasGRP3 expression in low-intensity inflammation.

## 1. Introduction

Inflammation is an important immune response to pathological conditions such as bacterial infection or tissue injury [1]. Host cells need to detect the intensity of infection and to respond properly to avoid excessive response [2]. Excessive or inadequate inflammation could lead to collateral tissue damage and various diseases [3]. For example, in case of influenza or coronavirus infection, uncontrolled release of inflammatory cytokines results in a cytokine storm that can lead to multiple-organ damage and even death [4,5].

Macrophages are important in the beginning and maintenance phases of inflammation. In the inflammatory response, macrophages play a role in phagocytosis, production of cytokines, and antigen presentation through major histocompatibility complex (MHC) molecules [6]. Since these cells are located in the brain, lungs, liver, kidneys, skin, intestines, vascular endothelium, testes, and blood [7], excessive activation of macrophages can affect the outcome of different organ systems in a variety of diseases. Hence, dysregulated activation of macrophages has been implicated in inflammatory diseases [6]. 

Pattern recognition receptors (PRRs) proteins are expressed on the membrane of immune cell, including macrophages. PRRs have four major sub-families: the Toll-like receptors (TLRs), the retinoic acid-inducible gene 1-like receptors, the nucleotide-binding oligomerization domain-Leucin Rich Repeats-containing receptors, and the C-type lectin receptors [8]. Fine tuning of PRRs’ signaling is involved in the detection and elimination of invading pathogens through pro-inflammatory signaling cascades related to the innate immune response [2]. Therefore, there are approaches that explore a negative regulation of PRRs’ signaling for proper inflammatory response.

Ras guanine nucleotide releasing protein 3 (RasGRP3), which is a guanine nucleotide exchange factor, is a negative regulator of the TLR-mediated response [2]. RasGRP3 negatively regulates TLR-induced pro-inflammatory cytokines to prevent excessive inflammatory response in low-intensity inflammation [2]. When RasGRP3 was silenced in macrophages, IL-6 was excessively produced by low-LPS stimulation [2]. In this context, RasGRP3 has attracted attention as a modulator of inflammation.

Citri Reticulatae Pericarpium is the peel of *Citrus reticulata* Blanco, which is commonly distributed on Jeju Island, Korea. Citri Reticulatae Pericarpium is a major medicinal source due to its therapeutic properties in Korean medicine; it has been traditionally used for a long time to treat various diseases. In addition, pharmacological studies have reported various therapeutic activities including anti-diabetes [9], anti-obesity [10], anti-cancer [11], and anti-inflammation [12] activities. 

In the present study, we investigated whether Citri Reticulatae Pericarpium extract (CRE) shows the regulatory activities of IL-6 overexpression on Raw264.7 macrophages. Its regulatory activity was significantly inhibited by the RasGRP3 gene silencing. Furthermore, we observed immunomodulatory effects of nobiletin, which is known as the major component of Citri Reticulatae Pericarpium [13,14], in low-intensity inflammation.

## 2. Results

### 2.1. Effects of CRE on the IL-6 Production in Raw264.7 Cells 

**Figure 1:**
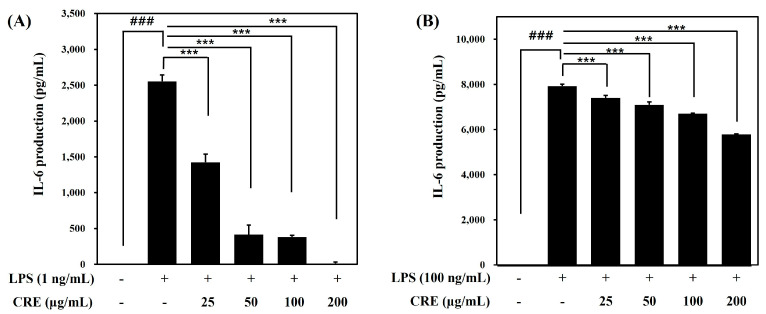
Effects of Citri Reticulatae Pericarpium extract (CRE) on the IL-6 production in Raw264.7 cells. Raw264.7 macrophages were treated with CRE (25, 50, 100, 200 μg/mL) 1 h before LPS treatment at a concentration of 1 ng/mL (**A**) or 100 ng/mL (**B**). The data are presented as mean ± SD. ^###^ *p* < 0.001 compared with the blank control group; and *** *p* < 0.001 compared with the LPS-treated group via one-way ANOVA (**A**) F(5, 12) = 3926, (**B**) F(5, 12) = 9149.

To assess whether CRE alleviates inflammation induced by LPS in Raw264.7 cells, the amount of IL-6 production was measured using an ELISA assay. The IL-6 production was significantly elevated by LPS treatment at a concentration of 1 and 100 ng/mL, compared with the untreated group (^###^ *p* < 0.001). As shown in Figure 1, the pretreatment with CRE (0, 25, 50, and 100 μg/mL) significantly suppressed the IL-6 production which was induced by 1 ng/mL and 100 ng/mL of LPS. In particular, the decrease in IL-6 production was stronger in the low-LPS environment (1 ng/mL) than in the high-LPS environment (100 ng/mL).

### 2.2. Effects of CRE on the RasGRP3 Expression in Raw264.7 Cells 

**Figure 2:**
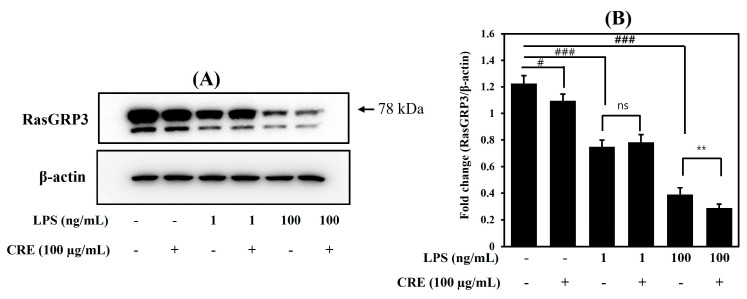
Effects of Citri Reticulatae Pericarpium extract (CRE) on RasGRP3 expression in Raw264.7 cells. Raw264.7 macrophages were treated with 100 μg/mL of CRE 1 h before LPS treatment at a concentration of 1 ng/mL (**A**) or 100 ng/mL (**B**). After 18 h post-LPS-treatment, the expression of RasGRP3 was determined using immunoblotting. ^#^ *p* < 0.05 ^###^ *p* < 0.001 compared with the blank control group; and ** *p* < 0.01 compared with the LPS-treated group.

The RasGRP3 expression was examined using immunoblotting. The treatment of 1 or 100 ng/mL LPS decreased RasGRP3 in Raw264.7 cells. The decrease in RasGRP3 was greater in the 1 ng/mL LPS group compared with the 100 ng/mL LPS group. The groups treated with CRE but not LPS showed no differences in RasGRP3 expression compared with the untreated group (Figure 2).

### 2.3. Effects of CRE on the IL-6 Production in Raw264.7 Cells Transfected with RasGRP3 siRNA 

**Figure 3:**
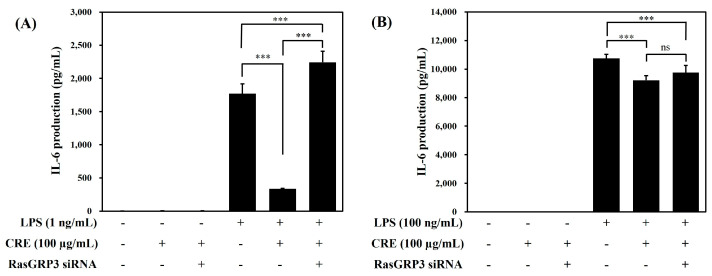
Effects of Citri Reticulatae Pericarpium extract (CRE) on the IL-6 production in Raw264.7 cells transfected with RasGRP3 siRNA. Raw264.7 macrophages with or without RasGRP3 siRNA were treated with 100 μg/mL of CRE 1 h before LPS treatment at a concentration of 1 ng/mL (**A**) or 100 ng/mL (**B**). The data are presented as mean ± SD. *** *p* < 0.001 compared with the LPS-treated group by one-way ANOVA (**A**) F (5, 18) = 491, (**B**) F(5, 18) = 1663.

We determined the effect of CRE on the IL-6 production using Raw264.7 cells transfected with siRNA against RasGRP3. The CRE pretreatment significantly suppressed IL-6 production induced by 1 ng/mL LPS; meanwhile, in the cells transfected with siRNA, this inhibitory effect of CRE on IL-6 production was not observed (Figure 3A). In the 100 ng/mL LPS group, CRE significantly inhibited IL-6 production; this inhibitory effect was not observed when cells were transfected with RasGRP3 siRNA (Figure 3B). 

### 2.4. Effects of CRE on the RasGRP3 Expression in Raw264.7 Cells Transfected with RasGRP3 siRNA

**Figure 4:**
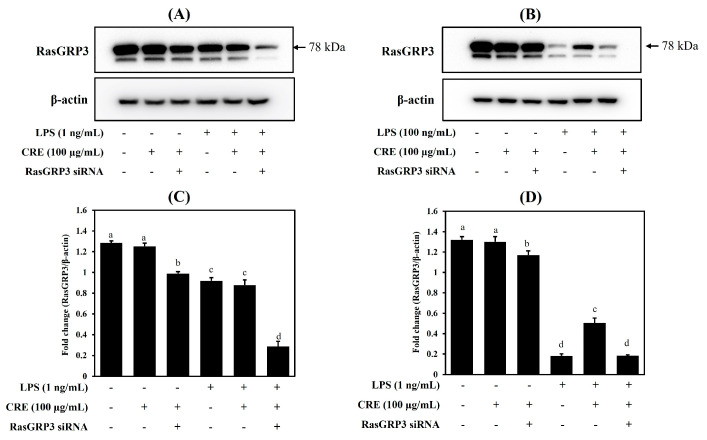
Effects of Citri Reticulatae Pericarpium extract (CRE) on the RasGRP3 expression in Raw264.7 cells transfected with RasGRP3 siRNA. Raw264.7 macrophages with or without RasGRP3 siRNA were treated with 100 μg/mL of CRE 1 h before LPS treatment at a concentration of 1 ng/mL (**A**) or 100 ng/mL (**B**). After 18 h post-LPS-treatment, the expression of RasGRP3 was determined using immunoblotting. Different letters (a–d) represent statistically significant differences (*p* < 0.05) (**C**,**D**).

To evaluate the effect of CRE on RasGRP3, we compared RasGRP3 protein expression between cells with or without transfection with siRNA against RasGRP3. In the low-LPS group (1 ng/mL), the expression of RasGRP3 was slightly decreased. There are no clear changes depending on LPS and CRE treatment in the low-LPS group (Figure 4A). As shown in Figure 4B, in the high-LPS group (100 ng/mL), CRE pretreatment upregulated the RasGRP3 expression decreased by LPS. This effect of CRE was not observed when the cells were transfected.

### 2.5. Effects of Nobiletin on the IL-6 Production in Raw264.7 Macrophages

**Figure 5:**
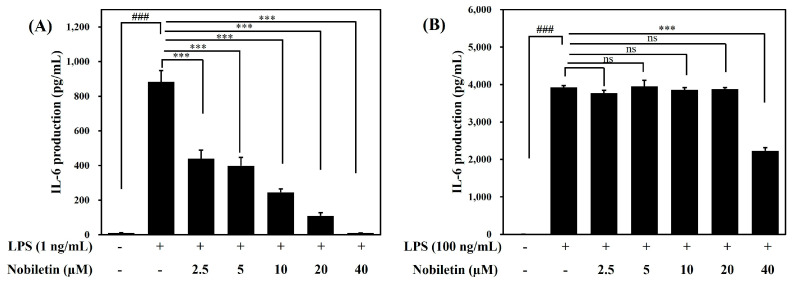
Effects of nobiletin on IL-6 production in Raw264.7 macrophages. Raw264.7 macrophages were treated with nobiletin (2.5, 5, 10, 20, 40 μg/mL) 1 h before LPS treatment at the concentration of 1 ng/mL (**A**) or 100 ng/mL (**B**). The IL-6 production was determined using a commercial IL-6 assay kit, following manufacturer’s instruction. The data are presented as mean ± SD. ^###^ *p* < 0.001 compared with the blank control group; and *** *p* < 0.001 compared with the LPS-treated group by one-way ANOVA (**A**) F (6, 21) = 278, (**B**) (6, 21) = 1335.

We examined an effect on IL-6 production of nobiletin, which is one of major components of Citri Reticulatae Pericarpium (442.6 ± 30.7 ppm, Figure S1 in Supplemental HPLC Data). As shown in Figure 5A, the pretreatment of nobiletin (2.5, 5, 10, 20, and 40 μM) significantly suppressed IL-6 production in the Raw264.7 cells treated with 1 ng/mL LPS (^###^ *p* < 0.001). However, pretreatment with nobiletin markedly reduced only at 40 μM against 100 ng/mL of LPS-induced IL-6 production (*** *p* < 0.001) (Figure 5B). In addition, pretreatment with nobiletin (at 20 μM or less) did not affect IL-6 production in cells treated with 100 ng/mL LPS (Figure 5B).

## 3. Discussion

Toll-like receptors (TLRs) are the most-investigated of the pattern recognition receptors (PRRs) [15]. TLRs recognize the structures of pathogens, and TLR signaling leads to an immune response, such as production of cytokines and type I IFNs, to eliminate pathogens [16]. To protect the host from overwhelming inflammation, there is a need for a negative regulator of TLR signaling. The previous study revealed that RasGRP3 limits excessive response in low-intensity inflammation [2].

Citri Reticulatae Pericarpium is a dried peel (exocarp, mesocarp, and endocarp) of *Citrus reticulata* Blanco, which is one of the most common and famous commercial citrus fruits in Korea. Besides its value as a fruit, its nutritional and therapeutic values have come to the fore. In particular, Citri Reticulatae Pericarpium has been used as a medicinal source in Korean medicine; therefore, various studies have been conducted to reveal its therapeutic effects. Recently, the immunomodulatory effect of Citri Reticulatae Pericarpium has been studied [17]. However, the effect of Citri Reticulatae Pericarpium on RasGRP3 has not been elucidated. The present study aims to demonstrate that Citri Reticulatae Pericarpium extract (CRE) has immunomodulatory effect via activating RasGRP3, using Raw264.7 macrophages.

Macrophages are the most notable immune cells. Under inflammation conditions, macrophages induce the pro-inflammatory cytokines, chemokines, and inflammatory mediators and enhance immune response via mobilizing immune cells [18,19]. Among the excessive cytokines induced by macrophages, IL-6 is one of the important [20]. 

In the present study, LPS treatment markedly increased IL-6 production in the Raw264.7 cells. The rise of IL-6 was greater in 100 ng/mL LPS-treated cells than in 1 ng/mL LPS-treated cells. Pretreatment with CRE suppressed IL-6 production; the decrease in IL-6 production was stronger in the low-LPS environment (1 ng/mL) compared with the high-LPS environment (100 ng/mL) (Figure 1). 

After this, we investigated the change of IL-6 production in cells transfected with siRNA against RasGRP3. In the 1 ng/mL LPS treated group, the effect of CRE on IL-6 production was significantly inhibited when cells were transfected with RasGRP3 siRNA (Figure 3A). However, in the 100 ng/mL LPS treated group, there was no significant change between groups with or without RasGRP3 siRNA transfection (Figure 3B). These results on IL-6 production imply that the CRE has regulatory effect on low-level inflammation, and we hypothesized that CRE may affect inflammatory response via activating RasGRP3.

To determine our hypothesis, the protein expression of RasGRP3 was observed. LPS treatment suppressed the expression of RasGRP3 proteins. The 1 ng/mL LPS treatment slightly decreased the RasGRP3 expression, but the 100 ng/mL LPS treatment markedly decreased it (Figure 2). In addition, we used the cells transfected with RasGRP3 siRNA to determine the effect of CRE on RasGRP3 expression. In the transfected cells with low-LPS (1 ng/mL), there were no clear changes depending on LPS and CRE treatment (Figure 4A). These results seem to be due to the mild inflammatory condition, when RasGRP3 expression slightly decreased compared with untreated cells. However, in the cells treated with high levels of LPS (100 ng/mL), CRE increased the expression of RasGRP3, and the increased RasGRP3 expression was blocked by RasGRP3 siRNA (Figure 4B). 

The pharmacological activities of medicinal herbs are related to their bioactive components. Polymethoxyflavones (PMFs) exclusively exist in citrus fruits and are one of the main components of citrus peel [21,22]. PMFs are flavonoids in citrus fruits that have been suggested to be beneficial to human health. It has been reported that citrus peel contains PMFs including sinensetin, nobiletin, heptamethoxyflavone, and tangeretin [22]. Nobiletin is an abundant PMF distributed in citrus fruit, including *Citrus reticulata* Blanco [13,14]. Previous studies revealed that nobiletin possess an anti-inflammatory effect [23,24]. In the present study, we studied the components of CRE, and we discovered that nobiletin has the most effective immune suppression activity (Figure 5).

In the present study, we observed that CRE has an immune hypersensitivity suppression effect on IL-6 production, and its effects appeared through the RasGRP3 protein. The immune hypersensitivity suppression effect was abolished with the transient RasGRP3 gene silencing method using siRNA.

The expression of RasGRP3 protein did not change in the low-LPS environment (1 ng/mL) compared with the untreated group, and CRE treatment did not increase RasGRP3 protein levels (Figure 4A). In a high-LPS environment (100 ng/mL), RasGRP3 expression was markedly suppressed, and this suppressed RasGRP3 expression was increased with CRE treatment (Figure 4B). However, increased RasGRP3 protein did not effectively reduce or alleviate IL-6 production in the high-LPS environment (100 ng/mL, Figure 3B). However, IL-6 production in the low-LPS environment (1 ng/mL) was significantly reduced with CRE treatment (Figure 3A). Moreover, the immune suppression effect of CRE in the low-LPS environment (1 ng/mL) was completely reversed with RasGRP3 siRNA transfection (Figure 3A). Additionally, the expression of RasGRP3 protein was also decreased with RasGRP3 siRNA transfection (Figure 4A). 

**Figure 6:**
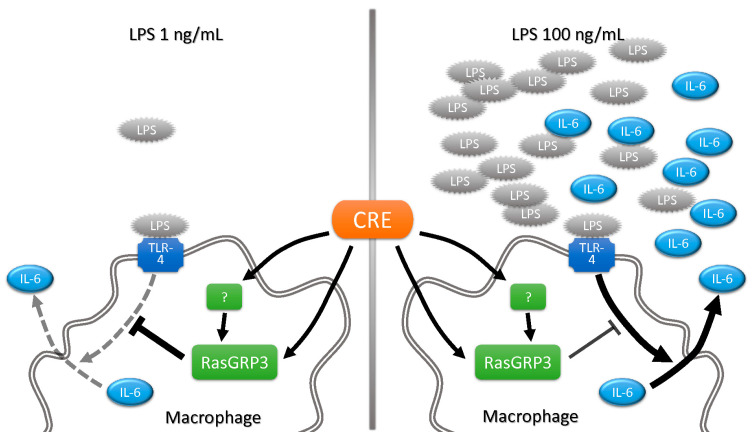
Proposed mechanism of immune hypersensitivity suppression activity of Citri Reticulatae Pericarpium extract (CRE) on the inflammatory responses induced by low-level (1 ng/mL) or high-level (100 ng/mL) LPS.

These results imply that CRE could upregulate RasGRP3 protein, but it is difficult to conclude that the RasGRP3 protein alone results in the immune hypersensitivity suppression effect of CRE. The proposed mechanism of the present study is shown in Figure 6. LPS triggers the activation of TLR responses and leads to the production of pro-inflammatory cytokines, such as IL-6. In a high-LPS environment, IL-6 production is more elevated compared with a low-LPS environment. To protect the host from the overwhelming production of cytokines, RasGRP3 plays the role of negative regulator in low-intensity inflammation [2]. CRE seems to alleviate hypersensitive immune responses through not only RasGRP3 but also other factors. Therefore, further research is needed to reveal details about the mechanisms of action of CRE. In addition, there are some limitations in our study. We only focused on LPS-induced RasGRP3 activation and successively increased IL-6 secretion. RasGRP3 limits pro-inflammatory cytokine production in macrophages by activating Rap1 protein [2]. Thus, the successive cascades of RasGRP3, including Rap1, should be further studied. Additionally, the mechanisms of nobiletin or other active components should be investigated.

Despite the unrevealed mechanism, our study showed that CRE has an immune hypersensitivity suppression effect via RasGRP3 on macrophages. These findings provide pharmacological evidence supporting the idea that that Citri Reticulatae Pericarpium has potential as a preventive agent to inhibit excessive inflammation and cytokine storm while eliminating pathogens. 

## 4. Materials and Methods

### 4.1. Chemicals

Antibody for RasGRP3 (#3334) was purchased from Cell signaling Technology (Beverly, MA, USA), and antibody for β-actin (SC-47778) was purchased from Santa Cruz Biotechnology (Santa Cruz, CA, USA). RIPA buffer, bovine serum albumin (BSA) protein kit, and ECL Western Blotting Substrate were obtained from Thermo Fisher Scientific. All other chemicals, of analytical reagent grade, were purchased from Sigma-Aldrich. The nobiletin, which was chosen from the previous papers [13,14], was also purchased from Sigma-Aldrich (chemical structure shown in Figure S1 in supplemental data).

### 4.2. Preparation of Citri Reticulatae Pericarpium Extracts (CRE)

Dried Citri Reticulatae Pericarpium was purchased from Omni Herb Inc. (Daegu, Republic of Korea). The identity of the samples was confirmed and deposited in the college of Korean Medicine in Semyung University. Citri Reticulatae Pericarpium was extracted in distilled water for 2 h at 100 ± 2 °C. After filtration, the extract was evaporated in an air vacuum and lyophilized by using a freeze dryer. The crude extract powder (yield 27.13%, *w*/*w*) was stored at −20 °C for study. 

### 4.3. Cell Preparation and siRNA Transfection

Raw264.7 cells (ATCC, Rockville, MD, USA) were maintained in Dulbecco’s modified Eagle’s minimum essential medium (DMEM) containing 10% *v*/*v* fetal bovine serum (FBS), 100 IU/mL penicillin, and 100 μg/mL streptomycin. The cells were incubated at 37 °C under 5% CO_2_ in an incubator. The toxicity of CRE was not observed in all tested concentrations, as shown in the supplemental data (Figure S2). We synthesized and selected two small interfering RNA (siRNA) duplexes (Bioneer, Daejeon, Republic of Korea) for the knockdown of RasGRP3 expression and control siRNA. For transient knockdown, 21-nucleotide sequences of siRNAs were synthesized (Mouse-RasGRP3-siRNA sense: GAGUCUGUGUUUCGAAACUTT; antisense: AGUUUCGAAACACAGACUCTT, control siRNA sense: UUCUCCGAACGUGUCACGUTT; antisense: ACGUGACACGUUCGGAGAATT). Both RasGRP3 and control siRNA duplexes were transfected into macrophages using lipofectamine RNAiMAX (Thermo Fisher Scientific., Waltham, MA, USA) according to the protocol. Forty-eight hours later, the efficiency of RNA interference was determined via Western blot assays. 

### 4.4. IL-6 Production in Raw264.7 Cells 

The Raw264.7 cells were transferred to 24-well plates (1 × 10^5^ cells per well) and pretreated with CRE dissolved in distilled water at concentration of 0, 25, 50, 100, 200 μg/mL. After 1 h of pretreatment, the cells were treated with LPS (1 or 100 ng/mL) and incubated for 20 h. Thereafter, IL-6 levels in supernatants were evaluated using a commercial IL-6 assay kit, following the manufacturer’s instruction. The nobiletin was dissolved with dimethyl sulfoxide (DMSO) and treated in 0.5% or less DMSO as a final concentration.

### 4.5. Western Blotting

Raw264.7 cells were washed with PBS and lysed using RIPA buffer. The concentration of protein was measured using a BCA assay kit. Sodium dodecyl sulfate (SDS) sample buffer (4×) was added to each lysed cell, and the samples were heated at 100 °C for 5 min. The samples were electrophoresed by SDS-PAGE and transferred onto nitrocellulose membrane. After 1 h blocking in 5% skimmed milk at room temperature, the membranes were incubated with primary antibodies (1:1000) of RasGRP3, β-actin during overnight at 4C. After incubation with secondary antibodies (1:5000) for 2 h at room temperature, signals were visualized with Western blotting luminal reagent and read on a Chemidoc (VILBER LOURMAT, Eberhaedzell, Germany).

### 4.6. Statistical Analysis

Data are expressed as mean ± standard deviations (SD). Statistical analysis was conducted with one-way ANOVA followed by Tukey multiple comparisons test post hoc analysis using GraphPad Prism (version 8.0.1, Dotmatics; La Jolla, CA, USA). Differences with *p* < 0.05 were considered statically significant.

## Data Availability

The data used to support the findings of this study are included in the article.

## Supplementary Materials

The following supporting information can be downloaded at: https://www.mdpi.com/article/10.3390/ijms241813777/s1

## References

[1] Hotamisligil G.S. (2006). Inflammation and Metabolic Disorders. Nature.

[2] Tang S., Chen T., Yu Z., Zhu X., Yang M., Xie B., Li N., Cao X., Wang J. (2014). RasGRP3 Limits Toll-like Receptor-Triggered Inflammatory Response in Macrophages by Activating Rap1 Small GTPase. Nat. Commun..

[3] Serhan C.N., Caterina R.D., Martinez J.A., Kohlmeier M. (2020). Chapter 61—Nutrients and Gene Expression in Inflammation. Principles of Nutrigenetics and Nutrigenomics.

[4] Li Z., Li L., Zhao S., Li J., Zhou H., Zhang Y., Yang Z., Yuan B. (2018). Re-Understanding Anti-Influenza Strategy: Attach Equal Importance to Antiviral and Anti-Inflammatory Therapies. J. Thorac. Dis..

[5] Coperchini F., Chiovato L., Croce L., Magri F., Rotondi M. (2020). The Cytokine Storm in COVID-19: An Overview of the Involvement of the Chemokine/Chemokine-Receptor System. Cytokine Growth Factor Rev..

[6] Shapouri-Moghaddam A., Mohammadian S., Vazini H., Taghadosi M., Esmaeili S.-A., Mardani F., Seifi B., Mohammadi A., Afshari J.T., Sahebkar A. (2018). Macrophage Plasticity, Polarization, and Function in Health and Disease. J. Cell. Physiol..

[7] Fujiwara N., Kobayashi K. (2005). Macrophages in Inflammation. Curr. Drug Targets-Inflamm. Allergy.

[8] Walsh D., McCarthy J., O’Driscoll C., Melgar S. (2013). Pattern Recognition Receptors—Molecular Orchestrators of Inflammation in Inflammatory Bowel Disease. Cytokine Growth Factor Rev..

[9] Ali A.M., Gabbar M.A., Abdel-Twab S.M., Fahmy E.M., Ebaid H., Alhazza I.M., Ahmed O.M. (2020). Antidiabetic Potency, Antioxidant Effects, and Mode of Actions of *Citrus Reticulata* Fruit Peel Hydroethanolic Extract, Hesperidin, and Quercetin in Nicotinamide/Streptozotocin-Induced Wistar Diabetic Rats. Oxid. Med. Cell. Longev..

[10] Guo X., Cao X., Fang X., Guo A., Li E. (2021). Inhibitory Effects of Fermented Ougan (*Citrus reticulata Cv. Suavissima*) Juice on High-Fat Diet-Induced Obesity Associated with White Adipose Tissue Browning and Gut Microbiota Modulation in Mice. Food Funct..

[11] Castro M.A., Rodenak-Kladniew B., Massone A., Polo M., de Bravo M.G., Crespo R. (2018). *Citrus Reticulata* Peel Oil Inhibits Non-Small Cell Lung Cancer Cell Proliferation in Culture and Implanted in Nude Mice. Food Funct..

[12] Jung K.H., Ha E., Kim M.J., Won H.-J., Zheng L.T., Kim H.K., Hong S.J., Chung J.H., Yim S.-V. (2007). Suppressive Effects of Nitric Oxide (NO) Production and Inducible Nitric Oxide Synthase (INOS) Expression by *Citrus Reticulata* Extract in RAW 264.7 Macrophage Cells. Food Chem. Toxicol..

[13] Li S., Wang H., Guo L., Zhao H., Ho C.-T. (2014). Chemistry and Bioactivity of Nobiletin and Its Metabolites. J. Funct. Foods.

[14] Nakajima A., Nemoto K., Ohizumi Y. (2020). An Evaluation of the Genotoxicity and Subchronic Toxicity of the Peel Extract of Ponkan Cultivar ‘Ohta Ponkan’ (*Citrus reticulata* Blanco) That Is Rich in Nobiletin and Tangeretin with Anti-Dementia Activity. Regul. Toxicol. Pharmacol..

[15] Lannoy V., Côté-Biron A., Asselin C., Rivard N. (2023). TIRAP, TRAM, and Toll-Like Receptors: The Untold Story. Mediat. Inflamm..

[16] Kondo T., Kawai T., Akira S. (2012). Dissecting Negative Regulation of Toll-like Receptor Signaling. Trends Immunol..

[17] Shin M.-S., Park S.B., Shin K.-S. (2018). Molecular Mechanisms of Immunomodulatory Activity by Polysaccharide Isolated from the Peels of *Citrus Unshiu*. Int. J. Biol. Macromol..

[18] Liu Y., Chen W., Zheng F., Yu H., Wei K. (2022). Xanthatin Alleviates LPS-Induced Inflammatory Response in RAW264.7 Macrophages by Inhibiting NF-ΚB, MAPK and STATs Activation. Molecules.

[19] Koh T.J., DiPietro L.A. (2011). Inflammation and Wound Healing: The Role of the Macrophage. Expert Rev. Mol. Med..

[20] Liu B., Li M., Zhou Z., Guan X., Xiang Y. (2020). Can We Use Interleukin-6 (IL-6) Blockade for Coronavirus Disease 2019 (COVID-19)-Induced Cytokine Release Syndrome (CRS)?. J. Autoimmun..

[21] Long T., Lv X., Xu Y., Yang G., Xu L.-Y., Li S. (2019). Supercritical Fluid CO2 Extraction of Three Polymethoxyflavones from Citri Reticulatae Pericarpium and Subsequent Preparative Separation by Continuous High-Speed Counter-Current Chromatography. J. Chromatogr. B.

[22] Zhang H., Tian G., Zhao C., Han Y., DiMarco-Crook C., Lu C., Bao Y., Li C., Xiao H., Zheng J. (2019). Characterization of Polymethoxyflavone Demethylation during Drying Processes of Citrus Peels. Food Funct..

[23] Wang H., Guo Y., Qiao Y., Zhang J., Jiang P. (2020). Nobiletin Ameliorates NLRP3 Inflammasome-Mediated Inflammation through Promoting Autophagy via the AMPK Pathway. Mol. Neurobiol..

[24] Ozkan A.D., Kaleli S., Onen H.I., Sarihan M., Eskiler G.G., Yigin A.K., Akdogan M. (2020). Anti-Inflammatory Effects of Nobiletin on TLR4/TRIF/IRF3 and TLR9/IRF7 Signaling Pathways in Prostate Cancer Cells. Immunopharmacol. Immunotoxicol..

