# Supplementary material for: Citri Reticulatae Pericarpium Limits TLR-4-Triggered Inflammatory Response in Raw264.7 Macrophages by Activating RasGRP3
Source: Int J Mol Sci. 2023 Sep 7;24(18):13777. doi: 10.3390/ijms241813777 (PMC10530606; doi:10.3390/ijms241813777)
Supplement: Supplementary file 1 [file ijms-24-13777-s001.zip › ijms-2576030-supplementary.pdf]

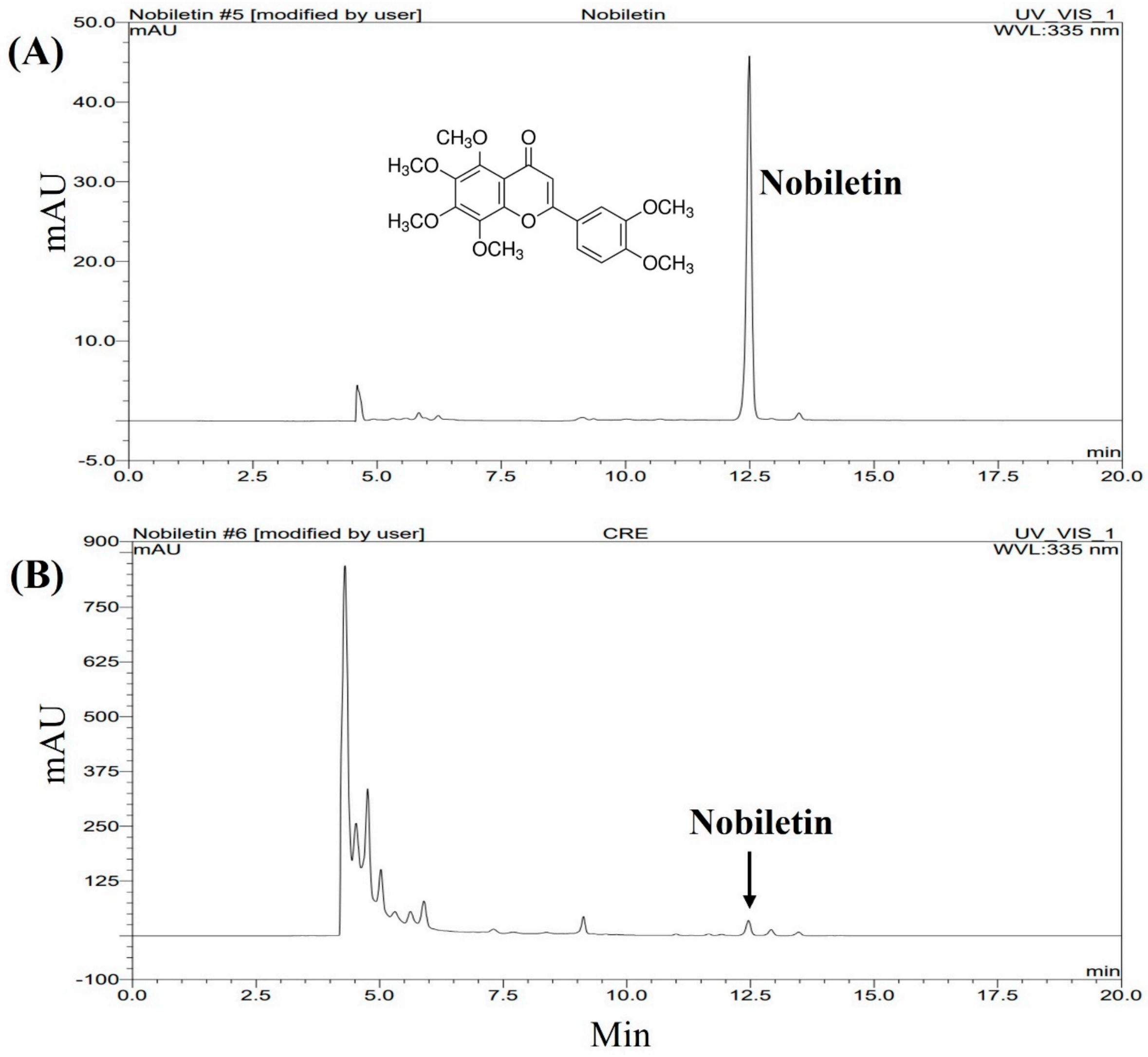

Figure S1. HPLC analysis graph.

We performed high-performance liquid chromatography (HPLC) to determined and quantify a nobiletin in the CRE. A Thermo Scientific (Ultimate 3000) HPLC system (Thermo Scientific, Korea) equipped with a UV detector and 20 μL injection loop was used. Chromatographic separation was carried out on a Thermo Scientific syncronis C18 columms (250×4.6 mm i.d., 5 μm). The flow rate was 0.8 mL/ min, and wavelength UV detection was set to 335 nm. Mobile phases were formed using 0.1% acetic acid in DW as eluent A and ACN as eluent B. The initial elution solution was 35% B, followed by a linear gradient to 55% B from 4 to 6 min, and a linear gradient to 70% B for 4 min. This proportion was maintained for 8 min. The mobile phase was immediately returned to the initial condition and maintained for 2 min until the end of the run.

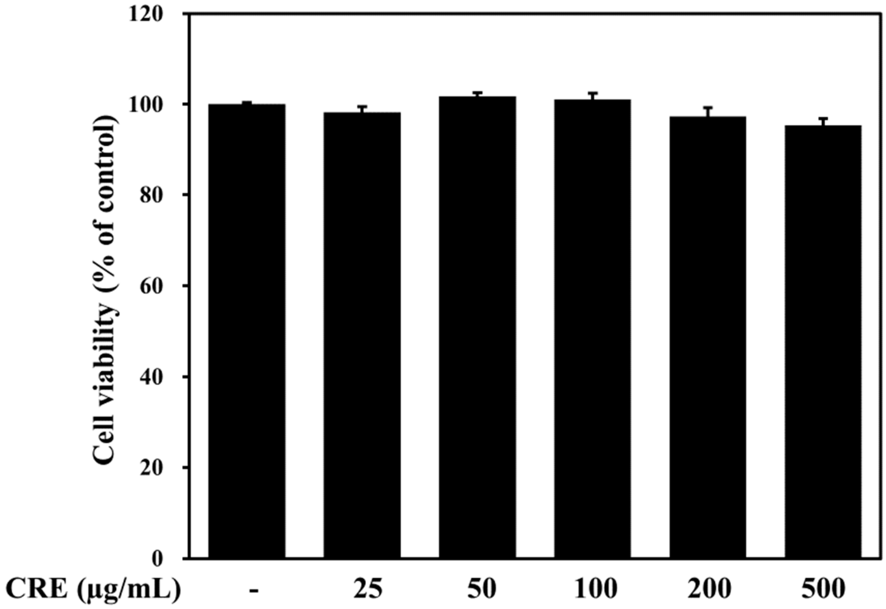

Figure S2. The cell viability results of Citri Reticulatae Pericarpium extract (CRE) in Raw264.7 cells. Raw264.7 macrophages were treated with 0, 25, 50, 100, 200, and 500 μg/mL CRE. The data were presented as mean ± SD.
